# A novel intraoperative continuous monitoring method combining dorsal cochlear nucleus action potentials monitoring with auditory nerve test system

**DOI:** 10.1186/s40463-023-00671-4

**Published:** 2023-10-06

**Authors:** Makoto Hosoya, Yuriko Nagaoka, Takeshi Wakabayashi, Marie N. Shimanuki, Takanori Nishiyama, Masafumi Ueno, Hiroyuki Ozawa, Naoki Oishi

**Affiliations:** Department of Otolaryngology-Head and Neck Surgery, Keio University School of Medicine, 35 Shinanomachi, Shinjuku-ku, Tokyo 160-8582 Japan

**Keywords:** Cochlear nerve monitoring, Cochlear implantation, Vestibular schwannoma

## Abstract

Highly accurate real-time cochlear nerve monitoring to preserve cochlear nerve function is essential for simultaneous cochlear implantation and ipsilateral vestibular schwannoma resection. In the present study, we developed a novel real-time monitoring system that combines dorsal cochlear nucleus action potential monitoring with intracochlear stimulating electrodes (Auditory Nerve Test System, ANTS). We used this system for a case with vestibular schwannoma resection via the translabyrinthine approach. The monitoring system developed in this study detected highly reliable evoked potentials from the cochlear nerve every two seconds continuously during tumor resection. Near-total tumor resection was achieved, and cochlear implantation was performed successfully after confirming the preservation of cochlear nerve function in a case. The patient’s hearing was well compensated by cochlear implantation after surgery. Our novel method continuously achieved real-time monitoring of the cochlear nerve every two seconds during vestibular schwannoma resection. The usefulness of this monitoring system for simultaneous tumor resection and cochlear implantation was demonstrated in the present case. The system developed in this study is compatible with continuous facial nerve monitoring. This highly accurate and novel monitoring method will broaden the number of candidates for this type of surgery in the future.

**Supplementary Information:**

The online version contains supplementary material available at 10.1186/s40463-023-00671-4.

## Background

Vestibular schwannoma is a clinically benign schwannoma that arises from the vestibulocochlear nerve, which runs through the internal auditory canal. Vestibular schwannomas account for approximately 8–10% of intracranial tumors and almost 80% of cerebellopontine angle tumors [1]. Audiological compensation using hearing devices is the only promising approach for treating hearing loss caused by vestibular schwannomas. Hearing devices include hearing aids, cochlear implants (CI), and auditory brainstem implants (ABI).

The simultaneous insertion of a cochlear implant and tumor resection is a feasible solution for hearing loss caused by vestibular schwannomas [2, 3]. When considering simultaneous cochlear implantation in ipsilateral vestibular schwannoma surgery, cochlear nerve preservation is essential, and cochlear nerve monitoring is required. Several factors are required for an ideal cochlear nerve monitoring. First, continuous real-time monitoring is preferable to intermittent monitoring during surgical procedures. Therefore, waveforms with higher amplitudes and shorter intervals should be generated using evoked responses. The waveforms must also be reproducible. Second, a monitoring method independent of cochlear implant electrodes is preferable. This is because it is preferable to open the cochlear implant after vestibular schwannoma resection and electrophysiological confirmation of the cochlear nerve preservation.

To date, several methods for monitoring the cochlear nerve during successful implantation of CI with vestibular schwannoma resection have been reported [4]. Conventional intraoperative auditory brainstem responses (ABRs), which are generated from sound stimuli via earphones inserted into the external ear canal, are widely used. However, this widely used conventional methods have several limitations. First, in most cases, patients with vestibular schwannoma experience a loss of wave V in the ABR. Second, ABR measurements require a relatively long time because of the requirement of averaging times. Therefore, this conventional method makes the precise real-time monitoring required in vestibular schwannoma surgery difficult to achieve.

Several methods for monitoring the cochlear nerves in such settings have been developed to overcome this limitation. For example, Patel et al. reported a method for monitoring with CI evoked electrical-ABR (E-ABR) and neural response imaging testing and achieved real-time auditory nerve monitoring [5]. Cochlear nerve action potential (CNAP) monitoring, in which the electrode is located on the cochlear nerve, is used for detecting evoked response, and more high amplitude waveforms in shorter intervals could be obtained compared conventional ABR; it has also been used for the cochlear nerve monitoring with vestibular schwannoma surgery with cochlear implantation [6].

Recently, an Auditory Nerve Test System (ANTS; MED-EL Corporation, Innsbruck, Austria) was developed [7, 8] with three components: an auditory nerve test electrode comprising an 18-mm electrode with three intracochlear contacts and an extracochlear ground, a stimulator box, and a connector cable. The stimulator box is connected to the CI programming hardware to send biphasic pulses to the electrode array, which can evoke E-ABR [8]. E-ABR confirmation during surgery with ANTS could lower the risk of opening a CI only for monitoring purposes, and E-ABR monitoring evoked by ANTS in vestibular schwannoma surgerywith simultaneous cochlear implantation has been reported [9, 10]. Kasbekar et al. reported a method for monitoring that combines this type of test electrode with an E-ABR. While in their report they tried to measure CNAP, but they failed to obtain a reliable waveform [11]. Recently, Sanna et al. also reported the usefulness of this type of intracochlear custom-made Med-El® test electrode and CNAP monitoring in vestibular schwannoma surgery with implantation of CI [6]. However, they measured the test electrode-evoked CNAP not in real-time but only after tumor resection to confirm cochlear nerve preservation.

In contrast to CNAP, dorsal cochlear nucleus action potential (DNAP) monitoring is another intraoperative method for vestibular schwannoma surgery, and its usefulness in preserving hearing has been reported [12–14]. To monitor DNAP, a detection probe was placed in the dorsal cochlear nucleus of the brainstem. The action potential of the dorsal cochlear nucleus is determined using this probe. DNAP is high sensitivity for electrical signals and can detect signals that ABR overlooks. Moreover, the DNAP system requires an average of only 100–200 stimulations. Intraoperative DNAP signals are obtained every 10 s. It has been reported that DNAP has higher stability in the surgical field and is more suitable than CNAP because of its electrode location [15].

We hypothesized that combining the ANTS with electrical DNAP (E-DNAP) would enable more accurate and sensitive intraoperative real-time continuous monitoring. In this report, we present an exprerience in a case of vestibular schwannoma resection with simultaneous implantation of CI using a novel intraoperative continuous monitoring system combining ANTS and DNAP measurement. In this case, we successfully obtained E-ABR and E-DNAP every two seconds and achieved good preservation of the cochlear nerve. The experience in this case will be useful for future intraoperative monitoring methods in planning vestibular schwannoma surgery with simultaneous implantation of CI.

## Our experience of the usage of the system

A 31-year-old female patient presented to our department with bilateral hearing loss and a vestibular schwannoma of the left ear. She had bilateral hearing loss since childhood and was using bilateral hearing aids since approximately two years of age. Her hearing loss progressed, and she adjusted her hearing aid at a previous hospital. However, she suffered from hearing loss, even with hearing aids, and was recommended cochlear implantation. She underwent magnetic resonance imaging (MRI) to investigate the possibility of CI, and a vestibular schwannoma on the left side was noted incidentally. The patient was then referred to our hospital.

**Fig. 1:**
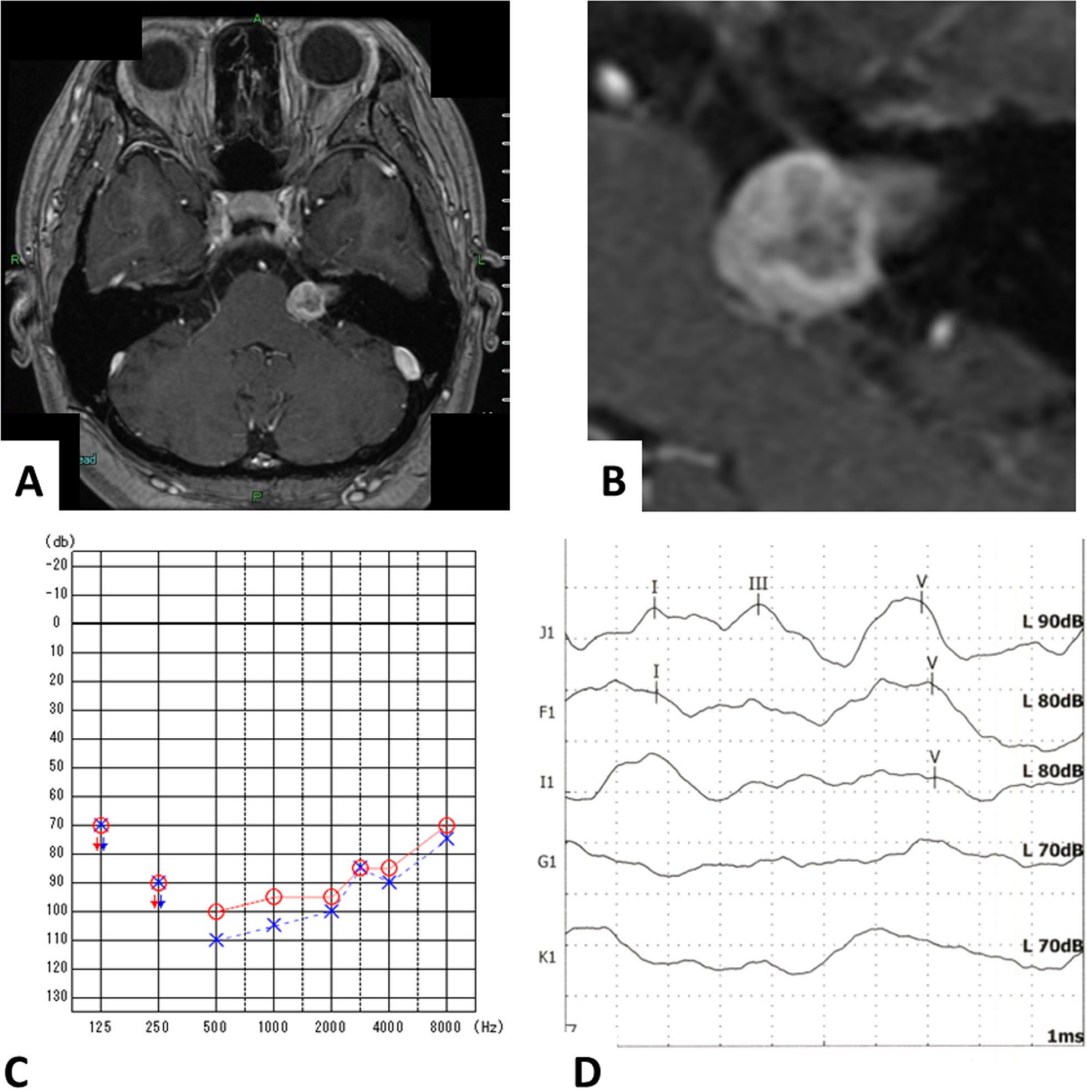
Preoperative MRI imaging and auditory test results. **A** Preoperative MRI showed a 23 mm well-defined, non-uniformly enhanced left internal ear canal tumor. **B** The tumor is distributed along the internal ear canal from the fundus to the cistern. **C**, **D** Preoperative audiological tests show bilateral profound hearing loss in the pure-tone audiometry (**C**), and only a slight wave V could be detected in the ABR in the left ear

MRI imaging revealed a well-defined, non-uniformly enhanced, 23 mm left internal ear canal mass distributed along the internal ear canal from the fundus to the cistern (Fig. 1A, B). On auditory examination, the patient showed profound hearing loss in both ears according to pure-tone audiometry (Fig. 1C). Her speech discrimination ability deteriorated in both ears, particularly on the left. A slight V-wave was observed in the ABR test (Fig. 1D). No obvious facial nerve paralysis was observed, whereas electroneurography showed a lower amplitude on the left side (11.5% in the orbicularis oculi muscle and 74.0% in the orbicularis oris muscle). At this point, we explained the possibility of simple resection of the vestibular schwannoma or simultaneous cochlear implantation with tumor resection. The patient finally chose simultaneous implantation of CI with vestibular schwannoma resection.

Internal auditory canal tumor resection was performed using a translabyrinthine approach. We planned to use a DNAP-electrode combined ANTS for precise cochlear nerve monitoring. We also planned to continuously monitor facial nerve function with facial nerve root muscle action potential (FREMAP) electrodes as facial nerve monitoring [16]. ANTS-evoked E-DNAP signals were collected using a CPAngle Master (Nihon Kohden, Tokyo, Japan), amplified, and filtered at 50–1500 Hz. The number of sweeps was 20. Biphasic pulses with a stimulation rate of 10.10 pulses per second (10.10 Hz) were used. Electrical stimulation was performed using a clinical Maestro system (MED-EL; Innsbruck, Austria).

**Fig. 2:**
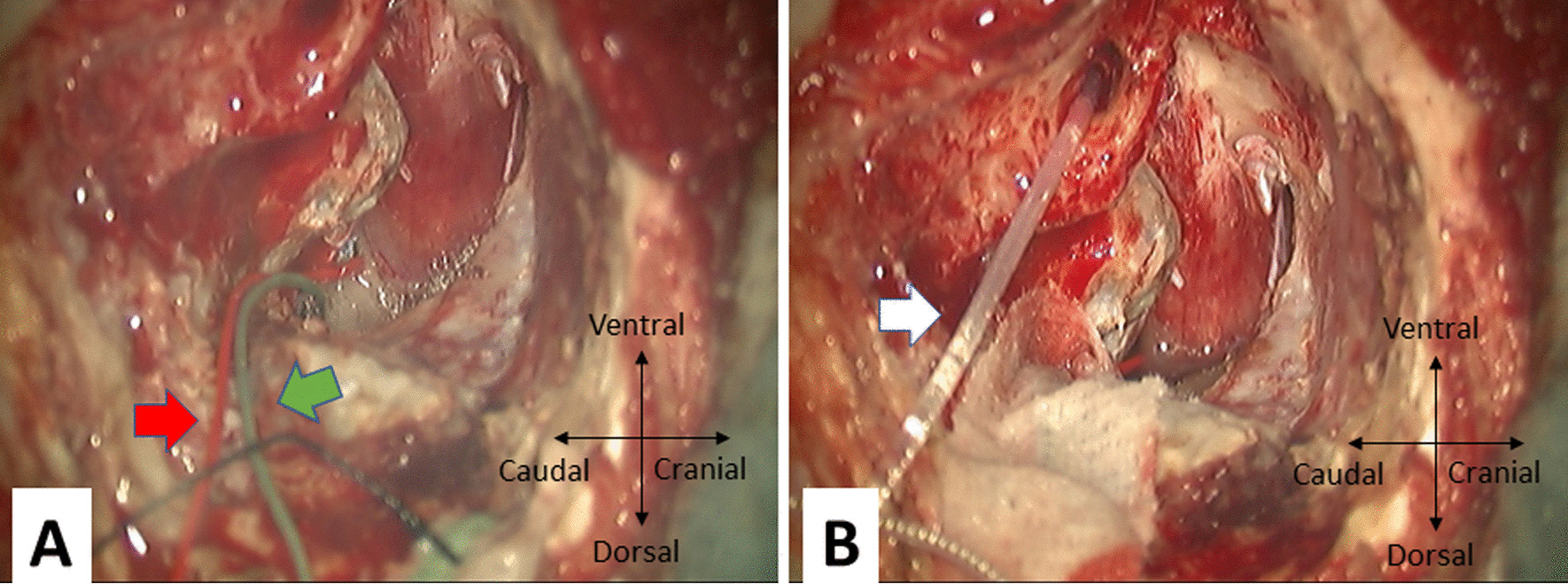
Intra-operative preparation of the FREMAP, DNAP, and ANTS electrodes. **A** After opening the cistern portion of the dura, FREMAP (Red Arrow), and DNAP (Green Arrow) electrodes were positioned before tumor resection. **B** ANTS electrode (White Arrow) was inserted into the cochlea via the round window

The round window niche was identified after cortical mastoidectomy and posterior tympanotomy. Subsequently, we performed a labyrinthectomy and reached the dura of the internal ear canal and posterior fossa. After opening the dura, the tumor was identified, and a FREMAP electrode was placed on the facial nerve root. The DNAP electrode was then placed on the dorsal cochlear nucleus (Fig. 2A). The ANTS electrodes were then inserted through the round window (Fig. 2B).

**Fig. 3:**
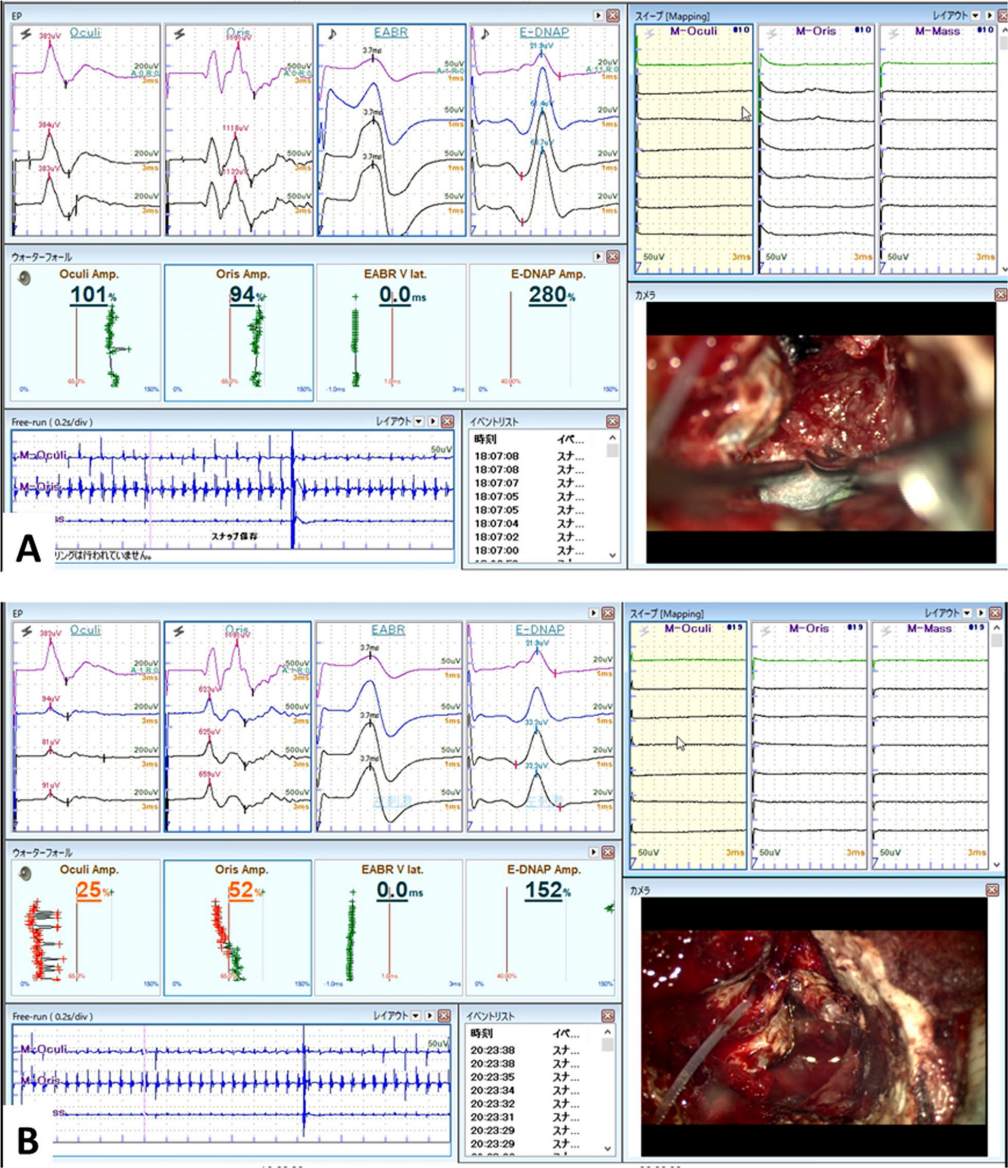
Intra-operative monitoring view. **A** FREMAPs were measured every three seconds during the tumor resection. ANTs-mediated E-ABR and E-DNAP are also detected every two seconds. If one of these waveforms deteriorated, the monitoring teams cautioned the surgeon. **B** Final monitoring results were shown. While FREMAP amplitude decreases were observed, EABR latency and EDNA amplitudes were maintained at the initial level

The ANTS generates synchronized electrical stimulations. E-ABR and E-DNAP were detected, as planned (Fig. 3) (Additional file 1: Video). Electrical pulses were generated using a Maestro system (10.10 Hz, Pulse Window 60.8usec). Subsequently, continuous E-ABR and E-DNAP monitoring were performed during tumor removal with continuous FREMAP monitoring.

After removing the cisternal portion of the tumor, it was carefully removed from the internal ear canal. Finally, we removed the tumor without deterioration of the ANTS-mediated E-ABR and E-DNAP and achieved nearly complete tumor removal (98%). The facial nerve function was also completely spared. During the operation, the amplitude of the E-DNAP gradually increased compared to its initial value once it reached approximately 400% of the initial amplitude. In comparison, it decreased to approximately 150% during tumor resection at the fundus of theinternal auditory canal. The final E-DNAP amplitude was > 200% (Fig. 3A). The FREMAP amplitude decreased to approximately 10% when the tumor was resected from the fundus of the internal auditory canal. Finally, the FREMAP amplitudes were 25% and 40% in the orbicularis oculi and orbicularis oris muscles, respectively (Fig. 3B).

**Fig. 4:**
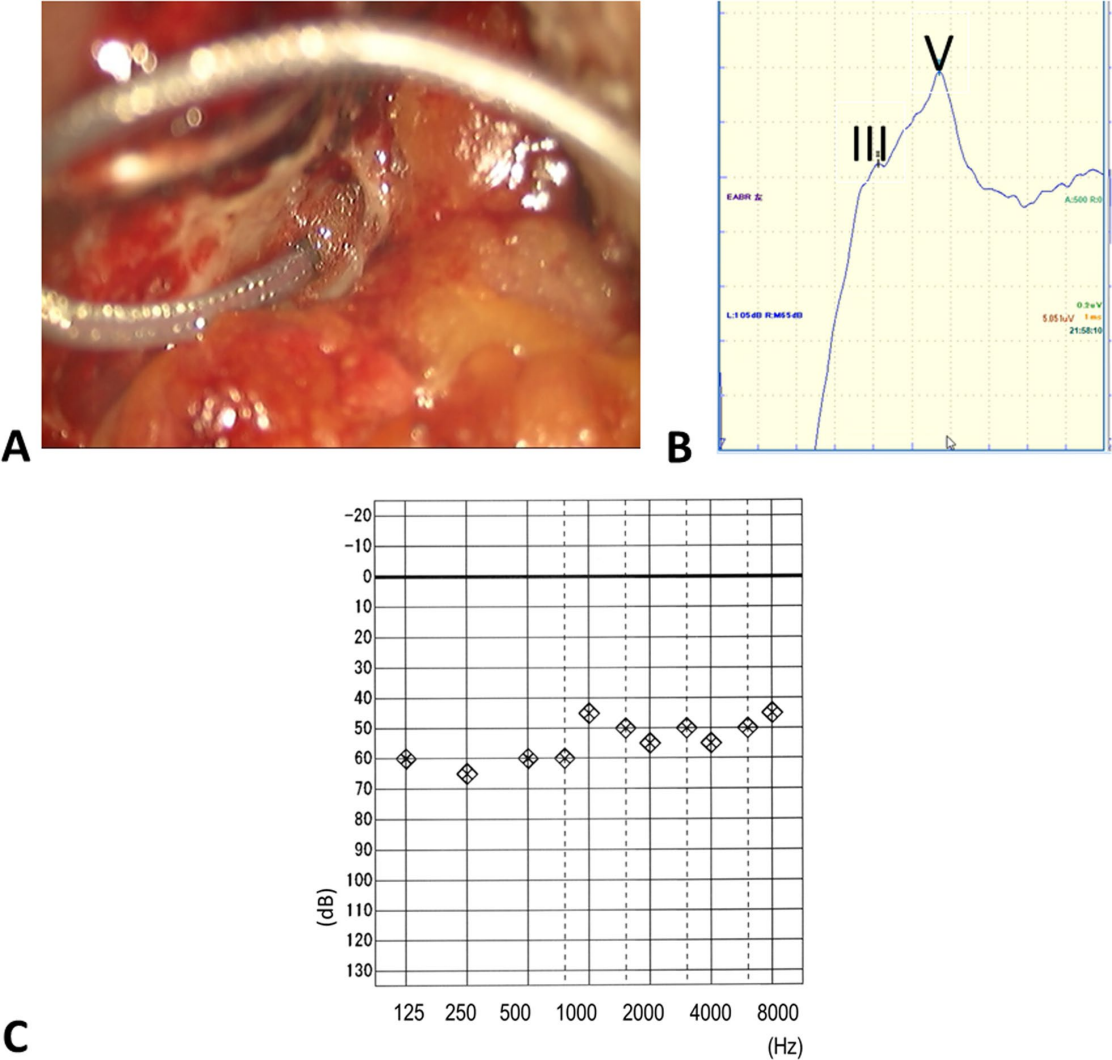
Insertion of the electrode of cochlear implantation and EABR measurement. **A** After tumor resection, the ANTS electrode was removed, and a cochlear implant electrode was inserted. **B** Well-defined EABR was also observed via cochlear implant stimulation. **C** After the surgery, the patient showed a improved hearing threshold

After confirming preservation of the electrophysiological function of the cochlear nerve, we carefully removed the ANTS electrode from the round window. We also simultaneously removed the FREMAP and DNAP electrodes. Subsequently, we inserted a CI electrode through a round window (FLEX 28 electrodes, MED-EL) (Fig. 4A). We measured the impedance of the implant electrodes and the NRT, which are usually measured in cochlear implantation surgery. The EABR through the CI was also measured at that time and clear EABR waveforms were detected (Fig. 4B). Finally, the open site of the dura was closed using the temporal muscle fascia and fibrin glue, and the operative cavity of the temporal bone was filled with abdominal fat.

The final histopathological report revealed schwannomas with a palisading pattern. Although slight left facial nerve palsy was observed after surgery (House-Brackmann Grade II), the patient fully recovered on postoperative day 13. No facial nerve paralysis was observed.

After discharge, we confirmed that the CI worked well. The CI improved her hearing threshold on the left side (Fig. 4C) and the patient showed good speech discrimination.

Ethics approval to report this case was obtained from the Ethics Committee of Keio University School of Medicine (approval number: 20200033). Written informed consent was obtained from the patients for the publication of anonymized information in this article.

## Discussion

In most cases, whether hearing loss was caused by the vestibular schwannoma itself or by an intervention, wearing a hearing aid is the easiest way to compensate for it [17]. However, the effect of hearing compensation by a hearing aid on vestibular schwannomas is limited because the hearing loss caused by vestibular schwannomas is a retro-cochlear type sensory neural hearing loss. Moreover, compensation with a hearing aid is less effective in patients with severe or profound hearing loss.

Another way to compensate for vestibular schwannomas in patients with severe-to-profound hearing loss is surgical intervention with tumor resection and simultaneous implantation of implantable auditory devices. Thus, among the available hearing devices, ABI and CI have been used in such situations.

Compared with CI, ABI has a unique advantage in vestibular schwannoma cases: ABI can bypass the cochlear nerve and restore hearing. Therefore, preservation of the cochlear nerve during tumor resection in patients with vestibular schwannomas is unnecessary. However, the hearing quality of the ABI is generally limited compared to that of the CI [18, 19] because it is difficult for the ABI to reproduce the audiological tonotopy of the sound that the CI can reproduce. CI is another possible hearing compensation method for patients with vestibular schwannomas who require resection. It can also be used in patients with severe or profound hearing loss caused by vestibular schwannomas. While it has the advantage of regaining a relatively high performance compared to the ABI, cochlear nerve preservation during surgery is essential for successful implantation of CIs with vestibular schwannoma surgery.

In patients with severe bilateral hearing loss accompanying vestibular schwannoma, ipsilateral CI is indicated at the same time as the removal of the vestibular schwannoma because non-concomitant CI surgery would be more difficult with intracochlear degeneration and fibrosis induced by surgical stimulation [20]. However, the need to preserve the cochlear nerve during removal of the vestibular schwannoma is a barrier to the simultaneous implantation of CIs. In cases of severe hearing loss on the operative side, the ABR response to the usual click sound stimulation is often lost or diminished. Despite the reliable preservation of inner ear nerve function, intraoperative cochlear nerve monitoring is highly challenging.

In the present study, we developed a novel monitoring method in addition to conventional intraoperative ABR [11, 12]. This method combines the ANTS (MED-EL) with DNAP monitoring. In this system, ANTS, an intracochlear electrical stimulation system using electrodes inserted into the cochlea was used, instead of sound stimulation, to perform continuous intraoperative monitoring of EABR and E-DNAP. Thus, the DNAP electrode can continuously evaluate action potentials by placing an electrode near the dorsal cochlear nerve nucleus and performing intracochlear electrical stimulation using an electrode inserted into the cochlea, instead of sound stimulation.

In this study, reliable E-DNAP was developed with relatively slow 10.1 Hz stimulation, while it has been reported that vestibular schwannoma causes the deterioration of evoking the action potential with high-rate stimulation [21, 22]. Under these conditions, we obtained reliable and continuous ANTS-mediated E-DNAP every two seconds (10.1 Hz stimulation, averaging 20 times), making real-time monitoring during tumor resection possible. The developed E-DNAP was quantitatively measured during surgery by comparing the initial amplitude prior to tumor resection. Moreover, it is compatible with FREMAP monitoring without any electrical contamination.

We report a case in which an auditory nerve tumor was removed using a translabyrinthine approach. Ipsilateral implantation of CI was performed using this monitoring system and a good postoperative CI effect was obtained. This is thought to expand the range of possibilities for simultaneous cochlear implantation with the removal of vestibular schwannomas. Moreover, the possibility of ipsilateral CI and modification of the approach to cochlear nerve preservation in vestibular schwannoma surgery in patients with severe hearing loss may expand if the cochlear nerve can be preserved with high accuracy.

This report demonstrates the successful use of a combination of DNAP monitoring and ANTS. However, the system has several limitations. First, this system requires the insertion of ANTS stimulation electrodes into the cochlea; therefore, this system would not be applicable to patients with intracochlear vestibular schwannomas. Second, the approach involves placing DNAP electrodes on the brainstem; thus, a vestibular schwannoma with a large cistern portion of > 30 mm is not preferable. Finally, cases with relatively large vestibular schwannomas that should be approached other than the translabyrinthine approach are not suitable. Therefore, the present system is more suitable for vestibular schwannoma cases with CI application, with spared intracochlear spaces for electrode insertion, and a relatively small cistern portion of the vestibular schwannoma (< 30 mm) that can be removed using the translabyrinthine approach.

Another limitation of this study is that only a single case could be included because of the limitation of the national insurance of application for cochlea implantation; principally, only bilateral severe hearing loss cases are covered in Japan, and no cochlear implantation is permitted for single-sided hearing loss, which is the most of the vestibular schwannoma cases. Therefore, the findings of this study should be replicated in other patients and at other centers in future studies.

## Conclusion

In this study, we developed a continuous and qualitative real-time monitoring system combining ANTS and DNAP systems. Vestibular schwannoma resection was achieved using this system, with simultaneous ipsilateral implantation of CI. In this system, the cochlear nerve function can be continuously measured every 2 s, with high reproducibility. After surgery, the cochlear nerve was functionally preserved, and the patient showed good hearing through the CI. This system could be useful for future patients with vestibular schwannoma who undergo tumor resection and CI. This system could also broaden the number of candidates for simultaneous implantation of CI in patients with vestibular schwannomas.

### Supplementary Information

**Additional file 1:** View of the monitoring system used in this study (Video). ANTs-mediated E-ABR and E-DNAP could be detected every two seconds.

## Data Availability

All data generated or analysed during this study are included in this published article.

## Abbreviations

ABI: Auditory brainstem implants
ABR: Auditory brainstem response
ANTS: Auditory nerve test system
CI: Cochlear implants
CNAP: Cochlear nerve action potential monitoring
DNAP: Dorsal cochlear nucleus action potential
E-ABR: Electrical auditory brainstem response
E-DNAP: Electrical dorsal cochlear nucleus action potential
FREMAP: Facial nerve root muscle action potential
MRI: Magnetic resonance imaging
